# Substance P enhances the proliferation and migration potential of murine bone marrow-derived mesenchymal stem cell-like cell lines

**DOI:** 10.3892/etm.2015.2291

**Published:** 2015-02-13

**Authors:** MARIA JOSE DUBON, KI-SOOK PARK

**Affiliations:** 1 Graduate School of Biotechnology, Kyung Hee University, Yong-In, Gyeonggi 466-701, Republic of Korea; 2 East-West Medical Research Institute, Kyung Hee University, Seoul 130-701, Republic of Korea

**Keywords:** substance P, OP9 cell, ST2 cell, bone marrow-derived mesenchymal stem cells, proliferation, migration

## Abstract

Due to the therapeutic characteristics of bone marrow (BM)-derived mesenchymal stem cells (MSCs), clinical trials are testing the use of autologous or allogeneic MSCs for the treatment of several conditions. These therapies require large numbers of MSCs and numerous studies are attempting to find substances that could enhance the egression of endogenous MSCs from the BM into the periphery and increase their proliferation *in vivo* and *in vitro*. It has been reported that substance P (SP) has the potential to increase the expansion of MSCs *in vivo* and to induce their mobilization from the BM into the periphery. The aim of the present study was to investigate the effects of SP on the migration and proliferation potential of two BM-derived MSC-like cell lines, ST2 and OP9. SP was found to induce the migration potential of ST2 cells *in vitro.* Furthermore, SP increased the proliferation of the MSCs cell line, OP9 cell line. Cyclin D1 expression was observed to increase in the OP9 cells, indicating the activation of the cell cycle in response to SP. The upstream signals involved in these phenomena have yet to be elucidated, although previous studies have proposed the activation of the extracellular signal-regulated kinase-1/2 and Wingless/β-catenin pathways as possible mediators of the cellular proliferation of human MSCs in response to SP. The present results therefore suggest that SP would facilitate the obtainment of higher numbers of endogenous MSCs from patients or donors and/or shorten the process of *in vitro* expansion that could cause the MSCs to undergo changes in their innate therapeutic characteristics prior to their use in therapy.

## Introduction

Bone marrow (BM)-derived mesenchymal stem cells (MSCs) are becoming a target for use in cell and gene therapy due to the fact that they possess several therapeutic advantages. MSCs can contribute to wound healing and tissue regeneration through their multiple differentiation capacity, immune modulation properties and ability to promote the neovascularization of ischemic tissues (1–4). Clinical and preclinical trials based on stem cell therapies by use of MSCs have reported that these cells can be beneficial in the treatment of several conditions, such as renal failure, myocardial infarction and bone and cartilage diseases (4–6). A great majority of these trials are based on the allogeneic or autologous transplantation of MSCs isolated from healthy donors or patients. This therapeutic approach requires the obtainment of high numbers of MSCs, which can be accomplished by the isolation of autologous MSCs and further expansion *in vitro*; however, studies have shown that culture expansion of MSCs can cause them to gradually lose their early progenitor properties, such as multipotency, proliferation potential and bone-forming efficiency (7). Efforts are therefore being made to find substances that can enhance the natural egress of MSCs from the BM into the periphery, which would facilitate the harvest of higher numbers of MSCs and increase their proliferation *in vitro* in order to shorten the expansion process. Eventually, in this way, the changes in the therapeutic properties of MSCs would be minimized prior to their clinical application.

A study by Hong *et al* (8), showed that substance P (SP), an 11-amino acid neuropeptide involved in pain perception, induced the mobilization of cluster of differentiation (CD) 29-positive MSCs from the BM into the periphery, which were found to participate in wound repair. In addition, the same study revealed that SP enhanced the proliferation of human MSCs *in vitro*, most likely through the activation of the extracellular signal-regulated kinase-1/2 (ERK-1/2) and T-cell factor/lymphoid enhancer-binding factor/β-catenin pathways (8). Furthermore, in a previous study our data showed an increase in the fibroblast-colony forming units (CFU-F) inside the BM two days after the injection of SP in mice, suggesting that the MSC population increases inside the BM in response to SP. Furthermore, the data demonstrated the ability of SP to modulate other essential characteristics of MSCs, such as their differentiation potential (9); however, the mechanisms involved in the SP-mediated mobilization and expansion of MSCs have yet to be elucidated.

Based on the aforementioned findings, SP could be considered as one of the substances that could be tested for its use to improve the isolation and *in vitro* expansion processes of MSCs in the development of stem cell therapies by use of these cells. In order to evaluate this possibility and to search for stable cell lines that could permit further investigation into the mechanisms involved in the SP-mediated effects on MSCs, the present study was designed to test the effects of SP on the proliferation and migration potential of two mouse BM-derived MSC-like cell lines, OP9 and ST2.

## Materials and methods

### Cell lines and culture

The OP9 cell line was purchased from the American Type Culture Collection (#CRL-2749; Manassas, VA, USA) and maintained in α-Minimum Essential Medium (1X, without ribonucleosides and deoxyribonucleosides; Gibco-BRL, Carlsbad, CA, USA) containing 10% heat-inactivated fetal bovine serum (FBS; Gibco-BRL), 2 mmol/l L-glutamine (Gibco-BRL), 1.5 g/l sodium bicarbonate (Gibco-BRL), 100 U/ml penicillin and 100 μg/ml streptomycin (P/S; Gibco-BRL). The ST2 cell line was obtained from the Riken Bioresource Center Cell Bank (Tsukuba, Japan) and maintained in RPMI-1640 (Gibco-BRL) supplemented with 10% FBS and 1% P/S. The two cell lines were incubated at 37°C in a humidified atmosphere containing 5% CO_2_. At 80% confluence, the cells were harvested using 0.25% trypsin/EDTA (Gibco-BRL) and sub-cultured at a ratio of 1:3 to 1:4. The media were changed every 3–4 days. Only cells in passages five to eight were used for the experiments.

### Cell counting

SP was obtained from EMD Millipore (#05-23-0600; San Diego, CA, USA) and was prepared with 5% acetic acid (Sigma-Aldrich, St. Louis, MO, USA). To evaluate the cytotoxicity of SP and its effect on the proliferation of the OP9 and ST2 cell lines, cell counting was performed. Cultured OP9 or ST2 cells were seeded in six-well plates at a density of 1.3×10^4^ or 5×10^3^ cells/well, respectively. After 24 h of incubation, the media were switched to control media (growth media containing 5% acetic acid (22 μm), a solvent of SP) or media containing 0.1, 1, 10, 100 or 300 nM SP for 72 h. In the case of the serum-free condition experiment, the cells were incubated for 18–24 h with serum-free growth media, following which the cells were treated with SP for 72 h. The cells were collected, stained with trypan blue and mounted on a Marienfeld-Superior hemocytometer (Paul Marienfeld GmbH & Co. KG, Lauda-Königshofen, Germany) for cell counting. The number of alive and dead cells was counted. Three replicates were analyzed for each concentration of SP tested.

### 5-Bromo-2′-deoxyuridine (BrdU) incorporation assay

OP9 or ST2 cells were seeded on fibronectin-coated coverslips (1 μg/ml) in 24-well plates at a density of 2.7×10^3^ cells/well or in six-well plates at a density of 5×10^3^ cells/well, respectively. The cells were incubated for 24 h before the treatment with SP. The cells were treated with control media (growth media containing the appropriate amount of solvent of SP) or media containing 1, 10 or 100 nM SP for 48 h. In the case of the serum-free condition experiment, the cells were serum-starved for 18–24 h, following which the cells were treated with SP for 48 h. The OP9 and ST2 cells were treated with 20 μM BrdU (Sigma-Aldrich) for the last 12 or 6 h of incubation, respectively. For the antagonist experiment, the OP9 cells were pretreated for 30 min with the neurokinin-1 (NK-1) receptor antagonist RP67580 (1 μM) (Tocris Bioscience, Bristol, UK). The cells were then treated with 10 nM SP in the presence or absence of RP67580 (1 μM) for 48 h. Following immunocytochemical staining for BrdU, images were captured using a Leica fluorescence microscope (Leica Microsystems GmbH, Wetzlar, Germany) and the number of total and BrdU-positive cells was counted.

### Immunocytochemistry

For immunofluorescence staining, the cells were fixed on coverslips with 4% paraformaldehyde in phosphate-buffered saline (PBS) for 10 min on ice. Following permeabilization with 0.2% Triton X-100 (USB Corp., Cleveland, OH, USA) and blocking solution treatment (5% non-fat milk in PBS with 0.1% Triton X-100) for 30 min at room temperature, the cells were incubated with mouse monoclonal anti-BrdU primary antibody (1:20, #11-170-376-001; Roche Diagnostics GmbH, Mannheim, Germany) for 1.5 h at room temperature. Subsequent to being washed three times with 1% non-fat milk in PBS with 0.1% Triton X-100, the cells were treated with Alexa 488 anti-mouse immunoglobulin G1 secondary antibody (Invitrogen Life Technologies, Carlsbad, CA, USA) for 45 min at room temperature. When necessary, actin was stained with phalloidin (Invitrogen Life Technologies). Finally, the samples were mounted using ProLong^®^ Gold Antifade mounting solution with DAPI (Invitrogen Life Technologies) and left to dry overnight prior to observation. The samples were then examined using a fluorescence microscope (Leica Microsystems Gmbh).

### RNA isolation and reverse transcription-quantitative polymerase chain reaction (RT-qPCR) analysis

Total RNA was extracted from cultured ST2 or OP9 cells using TRIzol™ reagent (Invitrogen Life Technologies) according to the manufacturer’s instructions. Samples of 5 μg total RNA were used for single strand cDNA synthesis using Superscript First-Strand cDNA Synthesis System (Invitrogen Life Technologies) according to the manufacturer’s instructions. After reverse transcription, RNA was degraded by *Escherichia coli* RNase H (Invitrogen Life Technologies). qPCR was performed in 20 μl reaction buffer containing 1.5 U i-Taq DNA polymerase (iNtRON Biotechnology, Seongnam, Korea), 1X PCR buffer (iNtRON), 2.5 mM each of dATP, dCTP, dGTP and dTTP (iNtRON) and 10 pM of each specific primer (NK-1: F 5′-TGGACTCTGATCTCTTCCCCAACA-3′ and R 5′-GGACCCAGATGACAAAGATGACCA-3′). Primers were purchased from Cosmo Genetech Co., Ltd. (Seoul, Korea). PCR products were electrophoretically separated on 1.5% (w/v) agarose gels (M.biotech, Inc., Hanam, Korea) and visualized after staining with RedSafe nucleic acid staining solution (iNtRON).

### Western blotting

Cells were treated with the correct amount of solvent of SP (5% acetic acid) or 10 nM SP for 0 or 24 h under normal medium conditions. To obtain the cell lysate, the cells were rinsed twice with ice-cold PBS and incubated with 400 μl 2X sodium dodecyl sulfate (SDS) loading buffer [100 mM Tris-Cl (pH 6.8), 4% (w/v) SDS, 0.2% (w/v) bromophenol blue, 20% glycerol and 200 mM β-mercaptoethanol] for 5 min at room temperature. The cell lysate was collected and denatured at 92°C for 10 min. Protein samples were subjected to 10% SDS-polyacrylamide gel electrophoresis. The separated proteins were transferred onto nitrocellulose membranes (Whatman; GE Healthcare Life Sciences, Munich, Germany). Subsequent to being blocked with 5% non-fat milk in 20 mM Tris-buffer containing 0.1% Tween-20 (TBS-T), the membranes were incubated with mouse monoclonal anti-cyclin D1 primary antibody (DCS-3, 1:800, #sc-20044; Santa Cruz Biotechnology, Inc., Santa Cruz, CA, USA) diluted with TBS-T buffer containing 5% non-fat milk overnight at 4°C. The membranes were then incubated with horseradish peroxidase-conjugated secondary antibodies at room temperature for 30 min. Target proteins were visualized using enhanced chemiluminescence detection (EMD Millipore). Band densities were measured using ImageJ software (National Institutes of Health (NIH), Bethesda, MD, USA). If required, membrane stripping was performed using Thermo Scientific™ Restore™ Western Blot Stripping Buffer (Thermo Fisher Scientific, Inc., Waltham, MA, USA) for 15 min at room temperature. The membranes were re-blotted by use of mouse monoclonal anti-α-tubulin antibody (1:10,000, #T5618; Sigma-Aldrich).

### Wound healing migration assay

ST2 cells were seeded on six-well plates and incubated until complete confluence was reached. The media were switched to serum-free growth media for 18 h. The confluent monolayer was then wounded using a 200-μl yellow pipette tip and washed twice with PBS to remove the floating cells. Treatment with 10, 100 and 300 nM SP was performed for 9 h. If necessary, pretreatment with the NK-1 receptor antagonist RP67580 (10 μM) (Tocris Bioscience) was applied for 30 min. Following incubation, the cells were fixed with 4% paraformaldehyde in PBS for 10 min at room temperature and stained with crystal violet solution (1% solution; Sigma-Aldrich) for 30 min. Cell culture images were captured using a light microscope and the wound area was measured with ImageJ software (NIH).

### Statistical analysis

Quantitative data are presented as the mean ± standard error of mean or standard deviation. An unpaired Student’s t-test was applied to evaluate differences between two groups. P<0.05 was considered to indicate a statistically significant difference. All statistical analyses were performed using GraphPad version 5.01 (GraphPad Software, Inc., La Jolla, CA, USA).

## Results

### SP induces the mobilization of ST2 cells

Due to the as yet unexplained difficulty in isolating and culturing murine MSCs, several stable MSC-like cell lines derived from mouse BM have been established that permit the *in vitro* study of the properties of MSCs. One of these cell lines, ST2, has been shown to possess the characteristic trilineage differentiation potential of MSCs along with their capacity to support the growth and differentiation of early B-lineage and T cells (10–13).

**Figure 1:**
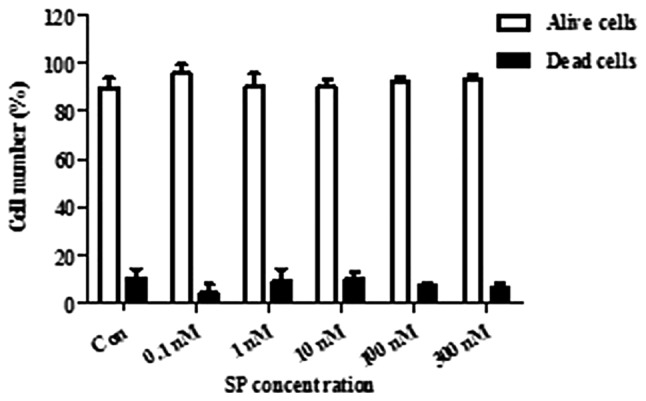
SP does not induce the proliferation of ST2 cells. Percentages of dead and alive cells following SP treatment. Cell viability was assessed on the basis of the exclusion of trypan blue-stained cells 72 h after SP treatment (0.1, 1, 10, 100 and 300 nM) under normal serum condition. Data are shown as the mean ± standard deviation (n=3 per group). Con, control; SP, substance P; BrdU, 5-bromo-2′-deoxyuridine.

**Figure 2:**
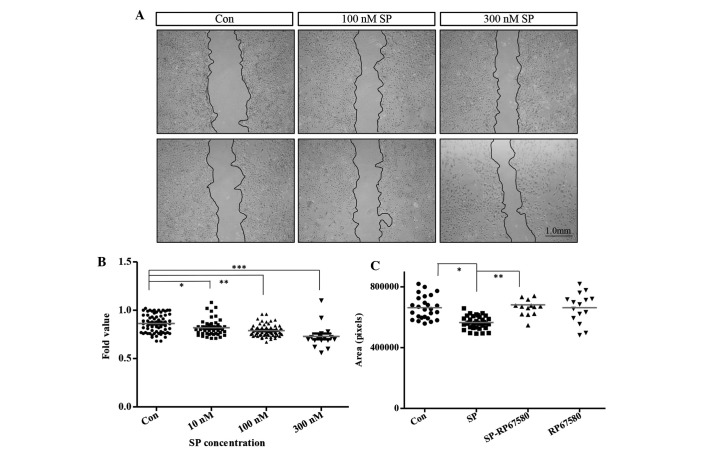
SP induces the mobilization of ST2 cells. (A) Representative images (scale bar, 1.0 mm) and (B) wound area measurement 9 h after SP treatment (10, 100 and 300 nM). Three independent experiments were performed and data are shown as the mean ± standard error of mean. Con (0 nM SP), n=68; 10 nM SP, n=45; 100 nM SP, n=70; 300 nM SP, n=17. ^*^P<0.0001, ^**^P<0.0148 and ^***^P<0.0001. (C) Wound area measurement 9 h after 300 nM SP treatment with or without 10 μM RP67580. Data are shown as the mean ± standard deviation. Con (0 nM SP), n=26; SP, n=35; SP-RP67580, n=14; RP67580, n=15. ^*^P<0.0001 and ^**^P<0.0001. Con, control; SP, substance P.

**Figure 3:**
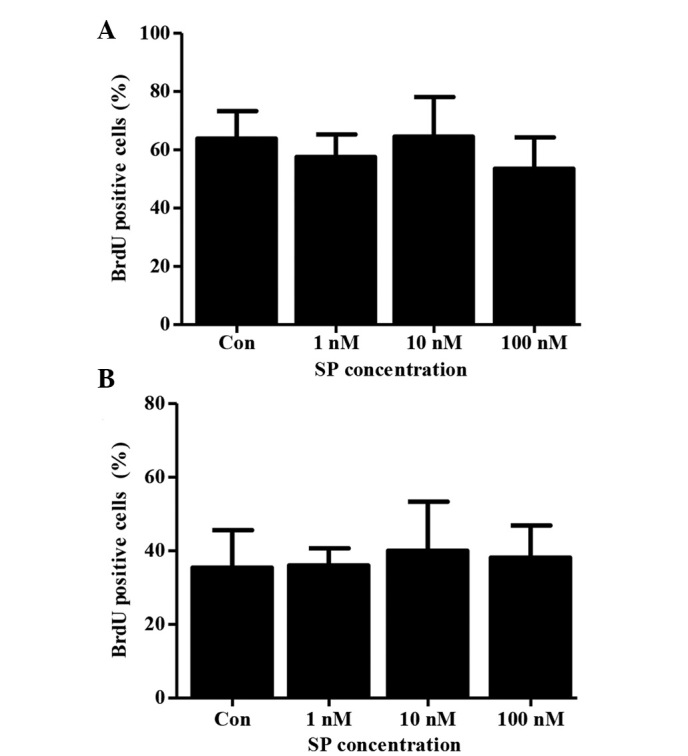
SP does not induce the proliferation of ST2 cells. Percentages of BrdU positive cells 48 h after SP treatment under (A) normal serum conditions or (B) serum-free conditions. Cells were treated with 20 μM BrdU for the final 6 h. The percentages of BrdU positive cells are presented as the mean ± standard deviation (n=3 per group). Con, control; SP, substance P; BrdU, 5-bromo-2′-deoxyuridine.

MSCs have been found to be able to mobilize in response to several signals from the BM into the periphery in order to contribute to the processes of wound healing and tissue repair (8). This results in an increase in the number of circulating MSCs, which could then be harvested and further expanded for their use in cell therapy. A previous study showed that SP induces the mobilization of MSCs *in vivo*; however, the mechanisms involved in this SP-mediated mobilization of MSCs have yet to be elucidated (8). To further analyze the effects of SP on the mobilization of MSCs, the effects of SP on the murine-derived stromal cell line ST2 were examined *in vitro*. The expression of the NK-1 receptor in ST2 cells was confirmed using RT-qPCR. No cytotoxic effect on the ST2 cells was observed following treatment with several concentrations of SP for 72 h (Fig. 1). A wound healing migration assay was subsequently performed in order to investigate the effect of SP on the mobilization of ST2 cells. It was found that SP induced the mobilization of ST2 cells at 9 h of SP treatment following the wound induction. This was reflected by a reduced wound area in the SP-treated groups compared with that in the untreated control group (Con) [Con, 0.86±0.01, n=68; 10 nM SP, 0.81±0.01 (P<0.0001 vs. Con), n=45; 100 nM SP, 0.78±0.006 (P<0.0148 vs. Con), n=70; 300 nM SP, 0.72±0.02 (P<0.0001 vs. Con), n=17] (Fig. 2A and B). Treatment with the NK-1 receptor antagonist RP67580 inhibited the migration induced by SP in the ST2 cells [Con, 662,300±14,930, n=26; SP, 565,100±103 (P<0.0001 vs. Con), n=35; SP-RP67580, 681,100±22,460 (P<0.0001 vs. SP), n=14; RP67580, 662,500±25,530, n=15] (Fig. 2C). To exclude the possibility that the reduction in the wound area in response to SP could be due to an enhanced proliferation of the cells, the effect of SP on the proliferation of ST2 cells was evaluated. No increase in the proliferation of this cell line was found in response to SP under normal or serum-free culture conditions following treatment with several concentrations of SP for 48 h (Fig. 3A and B). These data show that SP can induce the migration of ST2 cells and suggest that this cell line could be used in order to study the mechanisms involved in the SP-mediated mobilization of MSCs.

### SP increases the proliferation of OP9 cells under normal serum culture conditions

OP9, another important cell line that is often used to study the characteristics of MSCs, was established from newborn calvaria of the (C57BL/6 × C3H) F_2_-op/op mouse. By examining the immunophenotype, triple-differentiation capacity and immunological and migration features of these cells, they were found to be identical to standard MSCs. This cell line has been used in co-culture systems with mouse embryonic stem cells where they were found to support hematopoiesis (14).

**Figure 4:**
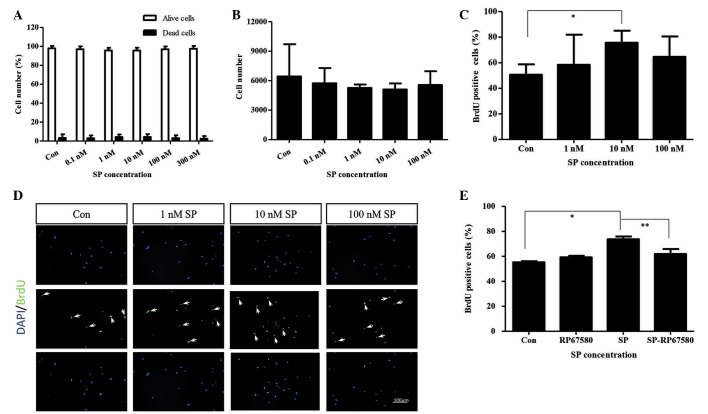
SP enhances the proliferation of OP9 cells. (A) Percentages of dead and alive cells 72 h after SP treatment (0.1, 1, 10, 100 and 300 nM). Cell viability was assessed on the basis of the exclusion of trypan blue-stained cells. Data are presented as the mean ± standard deviation (n=3 per group). (B) Numbers of OP9 cells 72 h after SP treatment (0.1, 1, 10 and 100 nM) under serum-free condition. Data are shown as the mean ± standard deviation (n=3 per group). (C) Percentages and (D) representative images of BrdU-positive cells 48 h after SP treatment (1, 10 and 100 nM) under normal serum conditions. Cells were treated with 20 μM BrdU for the last 12 h. Data are shown as the mean ± standard deviation. (n=5 per group). ^*^P<0.0021. Arrows indicate BrdU-positive cells. Nuclei were stained in blue with DAPI and BrdU was stained in green (scale bar, 100 μm). (E) Percentage of BrdU-positive cells 48 h after 10 nM SP treatment with or without 1 μM RP67580. Cells were treated with 20 μM. BrdU for the last 12 h. Data are shown as the mean ± standard error of mean from four independent experiments (n=4 per group). ^*^P<0.0002 and ^**^P<0.042. Con, control; SP, substance P; BrdU, 5-bromo-2′-deoxyuridine.

In a previous study, our data suggested that SP increased the number of MSCs inside the BM *in vivo*; therefore, an aim of the present study was to evaluate whether SP had the same effect on the proliferation of MSCs *in vitro* (9). Since no effect of SP was observed on the proliferation of ST2 cells, a decision was made to evaluate the effect of SP on another mouse-derived MSC line, knowing that established cell lines from the same cell type often present different characteristics due to their distinct origin. The effect of SP on the proliferation of the mouse-derived MSC line OP9 was therefore examined (Fig. 4). By performing RT-qPCR analysis the expression of the NK-1 receptor in the OP9 cells was confirmed (data not shown). No cytotoxic effect of SP on the OP9 cells was observed at any of the concentrations tested after 48 h of treatment (Fig. 4A). An increase in the proliferation of OP9 cells, which was reflected by higher numbers of BrdU-incorporating cells, was observed in the SP-treated groups compared with the control group after 72 h of treatment with different concentrations of SP; however, this increase was significant only at a concentration of 10 nM SP (Con, 50.6±3.6, n=5; 10 nM SP, 75.6±4.2, n=5; P<0.0021) (Fig. 4C and D). The specificity of this effect was then confirmed by treatment with the NK-1 receptor antagonist RP67580. It was observed that the effect of SP on the proliferation of OP9 cells was inhibited in the group treated with RP67580 in combination with SP compared with the group treated with SP only (SP, 74.5±1.6, n=4; SP-RP67580, 61.8±4.0, n=4, P<0.042) (Fig. 4E). It was of note that the effect of SP on the proliferation of OP9 cells was not observed in serum-free culture conditions, even after 72 h of treatment with increasing concentrations of SP (Fig. 4B). In combination, these data show that SP increases the proliferation of OP9 cells through the interaction with its receptor NK-1, and that this effect may require the presence of certain serum component(s).

### SP increases the levels of cyclin D1 in OP9 cells

**Figure 5:**
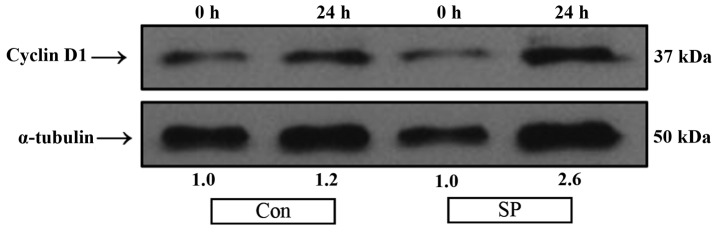
Protein expression of cyclin D1 increases with SP treatment in OP9 cells. Western blotting for the protein expression of cyclin D1 was performed with samples of total protein lysate extracted from OP9 cells after 0 or 24 h of treatment with 10 nM SP. Fold values were calculated following normalization with α-tubulin. Con, control; SP, substance P.

A key event in the activation of the cell cycle by proliferative signaling pathways is the activation of cyclin D1. An increased expression level of this protein is required for the G_1_- to S-phase transition of the cell cycle (15). In the present study it was evaluated whether the levels of cyclin D1 were altered by SP treatment in the OP9 cells. An increase in the levels of this protein was observed after 24 h of treatment in the SP-treated cells compared with the untreated control cells (Fig. 5). These data show that SP increases the levels of cyclin D1 in OP9 cells, suggesting that SP may promote the proliferation of OP9 cells by inducing the transition from G_1_- to S-phase of the cell cycle; however, further studies are required in order to prove this hypothesis and to evaluate the upstream signals involved in the SP-induced proliferation of OP9 cells.

## Discussion

The present study showed the ability of the neurotransmitter SP to enhance the migration potential of the mouse BM-derived MSC-like cell line ST2. SP was also demonstrated to increase the proliferation of another mouse BM-derived MSC-like cell line, OP9, under normal serum culture conditions. Furthermore, SP increased the level of cyclin D1 protein in the OP9 cells. The effects of SP on the proliferation and migration potential of the above-mentioned cell lines resulted from the interaction of SP with its receptor NK-1, which is expressed by these cells.

As more therapeutic properties and clinical applications of MSCs are being identified, there is an increasing requirement to improve the techniques to harvest endogenous BM-derived MSCs and to further expand them *in vitro*. This is important to contribute to the progress of the development of stem cell therapies based on the use of autologous or allogeneic BM-derived MSCs, as this would ultimately shorten the culture expansion process, which has been proven to influence the innate therapeutic characteristics of MSCs as early as the first passage culture (7,16–18). Reducing such changes could determine the successful application of MSCs under different therapeutic settings where the most innate stem cell properties are required. Alternatively, the success of the application of MSCs in certain therapeutic treatments could depend predominantly on the number of cells required to obtain a positive effect. At least three different approaches could be evaluated in the matter of improving the usage of MSCs as stem cell therapy: i) To increase the proliferation of endogenous MSCs *in vivo*; ii) to enhance the migration of MCSs from the BM into the periphery; and iii) to induce the proliferation of harvested endogenous MSCs *in vitro*. Accordingly, there is currently a search for substances that could stimulate the naturally occurring process of proliferation and liberation of endogenous reparative MSCs in the organism and/or alternatively induce higher expansion rates of MSCs *in vitro* (19).

The BM has been shown to be abundantly innervated with sensory nerves that, in addition to conducting information about different stimuli that could have the potential to cause tissue damage, also secrete a variety of neurotransmitters, such as SP and the calcitonin gene-related peptide, which could modulate the characteristics of the BM-derived stem cells (20). In a previous study we found that SP increased the CFU-F inside the BM two days after the injection of 5 nmol/kg SP in mice, suggesting that SP could increase the MSC population inside the BM and, in this way, allow the isolation of more MSCs from patients or donors (9). Further studies are required to correctly identify whether these cells are true MSCs, since the CFU-F in the BM comprises diverse cell populations.

Substances that can induce the mobilization of MSCs from the BM into the periphery to facilitate the harvest of higher numbers of endogenous MSCs are a current research focus; however, despite the fact that several substances, such as granulocyte-colony stimulating factor and AMD3100, have been suggested to induce an increase in the number of circulating MSCs, there is still no adequate protocol or drug combination that can give high yields of mobilized endogenous MSCs. This shows the necessity of expanding these cells *in vitro* in order to reach sufficient cell numbers for each treatment (19,21,22).

A study by Hong *et al* (8) proposed that SP could be a suitable stem cell mobilizer that could induce the mobilization of MSCs from the BM into the periphery. In the study it was shown that SP treatment caused an increase in the number of circulating CD29-positive MSCs, which were found to participate in wound repair; however, the mechanism for the SP-induced mobilization of MSCs remains unknown. This motivated us to search for a stable cell line that could permit the study of the mechanisms involved in the SP-mediated mobilization of MSCs. For this reason, the effect of SP on the migration potential of the ST2 cell line was evaluated in the present study. It was found that SP increased the migration potential of the ST2 cells 9 h after treatment in a concentration-dependent manner without affecting their proliferation potential. Furthermore, treatment with the NK-1 receptor antagonist RP67580 inhibited the migration induced by SP in the ST2 cells. These data support the findings obtained in the study by Hong *et al* (8). In our previous study, it was observed that SP increased the mRNA levels of N-cadherin and stromal cell-derived factor 1 inside the BM one day after the injection of 5 nmol/kg SP into mice, suggesting that these molecules could be involved in the mechanism by which SP induces the mobilization of MSCs (9); however, further studies are required to identify the role of these two molecules in the SP-mediated mobilization of MSCs and the migration of ST2 cells. In combination, these data show that SP enhances the migration potential of the mouse-derived MSC-like cell line ST2 and suggest that this cell line is suitable to investigate the mechanism(s) involved in the SP-mediated mobilization of MSCs.

A previous study demonstrated that SP induced the proliferation of human MSCs *in vitro* (8), as well as the activation of the ERK-1/2 pathway and an increase in the nuclear translocation of β-catenin (8); however, the question still remains of whether these pathways are involved in the mechanism(s) underlying the SP-mediated proliferation of MSCs. Having found that SP induced the proliferation of mouse MSCs *in vivo* (9), we subsequently evaluated whether SP could exert the same effects on the mouse BM-derived MSC-like cell line OP9 *in vitro* in order to evaluate the ability of SP to improve the *in vitro* expansion of MSCs and to examine the possibility of using this cell line to identify the mechanism(s) involved in the proliferation mediated by SP in MSCs. It was found that SP induced the proliferation of OP9 cells under normal serum culture conditions at an optimal concentration of 10 nM. An increase in the protein levels of cyclin D1 in the OP9 cells following treatment with 10 nM SP was also observed. Cyclin D1 is proposed to act as an active switch in the regulation of continued cell cycle progression, and high levels of this protein are known to be necessary for cells to progress from the G_1_- to the S-phase of the cell cycle (15,23).

The results of the present study suggest that SP could have the potential to induce OP9 cells to undergo a G_1_-/S-phase transition, hence inducing them to proliferate; however, further investigations are required to elucidate the effects of SP on the cell cycle in OP9 cells and to identify the upstream signals involved in the SP-mediated proliferation of OP9 cells. In this matter, the first candidates to be evaluated would be the mitogen-activated protein kinase/ERK and the Wingless/β-catenin pathways. It is of note that SP did not have any effect on the proliferation of OP9 cells under serum-free conditions, which suggests that SP may require the presence of certain serum component(s) to execute its stimulating effect. We did not performed any further studies in this matter, but it would be interesting to determine whether SP is also dependent upon the collaboration of other molecules to induce its effects on MSCs *in vivo*. In conclusion, this study presents evidence that SP could be considered for use in the development of stem cell therapies based on MSCs as a substance that could facilitate the harvest of high numbers of endogenous MSCs from patients and/or donors and increase the *in vitro* expansion rates of endogenous MSCs prior to their use in stem cell therapy.
